# Germination and Early Seedling Development in *Quercus ilex* Recalcitrant and Non-dormant Seeds: Targeted Transcriptional, Hormonal, and Sugar Analysis

**DOI:** 10.3389/fpls.2018.01508

**Published:** 2018-10-22

**Authors:** M. Cristina Romero-Rodríguez, Antonio Archidona-Yuste, Nieves Abril, Antonio M. Gil-Serrano, Mónica Meijón, Jesús V. Jorrín-Novo

**Affiliations:** ^1^Department of Biochemistry and Molecular Biology, Agrifood Campus of International Excellence, University of Córdoba, Córdoba, Spain; ^2^Departamento de Química Biológica, Dirección de Investigación, Facultad de Ciencias Químicas, Universidad Nacional de Asunción, San Lorenzo, Paraguay; ^3^Centro Multidisciplinario de Investigaciones Tecnológicas, Dirección General de Investigación Científica y Tecnológica, Universidad Nacional de Asunción, San Lorenzo, Paraguay; ^4^Instituto de Agricultura Sostenible, Consejo Superior de Investigaciones Científicas, Campus de Excelencia Internacional Agroalimentario, Córdoba, Spain; ^5^Departamento de Química Orgánica, Facultad de Química, Universidad de Sevilla, Seville, Spain; ^6^Plant Physiology Lab, Department of Organisms and Systems Biology, Faculty of Biology, University of Oviedo, Oviedo, Spain

**Keywords:** *Quercusilex*, recalcitrant, germination, early seedling growth, phytohormone

## Abstract

Seed germination and early seedling development have been studied in the recalcitrant species *Quercus ilex* using targeted transcriptional, hormonal, and sugar analysis. Embryos and seedlings were collected at eight morphologically defined developmental stages, S0–S7. A typical triphasic water uptake curve was observed throughout development, accompanied by a decrease in sucrose and an increase in glucose and fructose. Low levels of abscisic acid (ABA) and high levels of gibberellins (GAs) were observed in mature seeds. Post-germination, indole-3-acetic acid (IAA), increased, whereas GA remained high, a pattern commonly observed during growth and development. The abundance of transcripts from ABA-related genes was positively correlated with the changes in the content of the phytohormone. Transcripts of the drought-related genes *Dhn3* and *GolS* were more abundant at S0, then decreased in parallel with increasing water content. Transcripts for *Gapdh*, and *Nadh6* were abundant at S0, supporting the occurrence of an active metabolism in recalcitrant seeds at the time of shedding. The importance of ROS during germination is manifest in the high transcript levels for Sod and Gst, found in mature seeds. The results presented herein help distinguish recalcitrant (e.g., *Q. ilex*) seeds from their orthodox counterparts. Our results indicate that recalcitrance is established during seed development but not manifest until germination (S1–S3). Post-germination the patterns are quite similar for both orthodox and recalcitrant seeds.

## Introduction

*Quercus ilex* subsp. *ballota* [Desf.] Samp. is the dominant tree in forest ecosystems over large areas of the Western Mediterranean Basin (Pulido et al., 2001; de Rigo and Caudullo, 2016). As a *Quercus* species, it produces recalcitrant seeds, which are shed, and germinate, at a relatively high-water content (Connor and Sowa, 2003; Pasquini et al., 2012; Joët et al., 2013; Sghaier-Hammami et al., 2016). They are susceptible to desiccation injury, and become inviable when stored for long periods (Pasquini et al., 2012). Problems associated with loss of viability during collection and, processing, and the short storage life of these seeds, can lead to serious difficulties with seed conservation and propagation.

A number of studies focused on germination, storage, desiccation sensitivity, and viability after storage of *Quercus* spp. seeds have been previously published (Bonner and Vozzo, 1987; Finch-Savage and Blake, 1994; Finch-Savage et al., 1996; Connor and Sowa, 2003; Goodman et al., 2005; Berjak and Pammenter, 2008; Ntuli et al., 2011; Liu et al., 2012; Pasquini et al., 2012; Xia et al., 2014; Pariona et al., 2017; Shi et al., 2017). However, specific differences between recalcitrant and orthodox species (Weitbrecht et al., 2011; Rajjou et al., 2012; Swigonska and Weidner, 2013), and the molecular bases of *Q. ilex* recalcitrance remain enigmatic. In a previous study of *Quercus robur* it was found that recalcitrance does not on accumulation of dehydrins, abscisic acid (ABA), or soluble sugars during seed maturation (Finch-Savage and Blake, 1994).

Development of orthodox seeds ends at the desiccation stage, when the dry seeds enter a quiescent phase prior to germination. Major changes that occur during seed maturation involve multiple cellular processes including gene expression reprogramming which is under strict hormonal control. These include: synthesis of storage, heat-shock, and osmoprotective proteins and carbohydrates, activation of antioxidant defenses, and reduction of metabolism (Angelovici et al., 2010). In contrast, recalcitrant seeds are able to germinate immediately after shedding, without a quiescent phase, and continuously maintain high metabolic activity (Berjak and Pammenter, 2013; Caccere et al., 2013; Parkhey et al., 2014).

A complex interacting set of phytohormones, including ABA, gibberellins (GAs), ethylene, brassinosteroids (BR), auxin, and cytokinins (CK), control seed germination. ABA and GA are the prominent inhibitor and promoter, respectively, of germination (Rajjou et al., 2012). The ABA/GA ratio is thought to regulate the metabolic transition required for germination, but this has been largely studied in orthodox seeds and poorly investigated in recalcitrant seeds. It has been proposed that precocious germination of recalcitrant seeds is because of low levels of ABA in the mature embryos (Farrant et al., 1993; Prewein et al., 2006).

High metabolic activity during germination is generally accompanied by an increase in reactive oxygen species (ROS) (especially H_2_O_2_) (Kranner et al., 2010; Roach et al., 2010). ROS can act as secondary messengers in signal transduction pathways, that control several processes, including germination (Oracz and Karpiñski, 2016). Also, the overproduction of ROS has been recognized as a main cause of the deterioration associated with loss of seed vigor (Jeevan Kumar et al., 2015). Therefore, antioxidant defense is required to reduce the level of ROS produced during imbibition and germination (Bailly et al., 2008). An increase in anti-oxidant metabolites (ascorbate and glutathione) and enzymes (APX, SOD, and CAT) during seed imbibition and germination has been described in different species of orthodox seeds (Gara et al., 1997; Tommasi et al., 2001; Bogdanović, 2008; Lee et al., 2010; Wojtyla et al., 2016) but data from studies of recalcitrant species are scarce.

In this study, we have targeted transcriptional, hormonal, and metabolic analyses of mature, and germinating seeds, and young seedlings of *Q. ilex*. The transcript profiles of eleven genes coding proteins involved in: desiccation tolerance (DHN3 and GOLS) (Nishizawa et al., 2008; Hanin et al., 2011), ABA-signaling regulation (OCP3, SKP1, and SDIR1) (Cutler et al., 2010), metabolism (FDH, GAPDH, RBLC, and NADH6) (Muñoz-Bertomeu et al., 2009; David et al., 2010) and oxidative stress (SOD1 and GST) (Giannopolitis and Ries, 1977; Schopfer et al., 1985) were determined. Our results contribute to understanding the physiological changes that take place during seed germination and seedling growth of recalcitrant species such as *Q. ilex.*

## Materials and Methods

### Plant Material

Acorns, in accordance with the maturity indexes described by Bonner and Vozzo (1987) and used by Pasquini et al. (2012), were harvested from 10 *Q. ilex* trees randomly selected (Cerro Muriano, province of Córdoba, Spain; 37°59′57.74″N, 04°46′57.93″W) in December 2010. Climate data can be found at https://es.climate-data.org/location/657770/. Undamaged acorns were sterilized by immersion in 2.5% sodium hypochlorite for 10 min, abundantly rinsed with water, and dried by air circulation at room temperature on filter paper.

### Seed Germination

Acorns were hand dehulled in order to perform a synchronized germination, as suggested by Liu et al. (2012) (Supplementary Figure S1). Germination was performed in darkness at 22°C for a total period of 216 h. Seed embryos or germinated seedlings, were collected at 0, 6, 12, 24, 48, 72, 144, and 216 h after imbibition (Supplementary Figure S2), and immediately frozen in liquid nitrogen and stored at −70°C. Three biological replicates were performed for each sampling time, each one containing ten-to fifty, depending on the analysis and sampling time, individual embryonic axes or germinated seedlings.

### Relative Water Content

The relative water content (RWC) was determined for all developmental stages (S0–S7) according to the following equation: RWC = [(FW–DW)/FW] × 100, where FW and DW are fresh and dry weight, respectively. FW and DW were measured using an analytical balance. Dry weight was obtained after sample drying in an oven at 70°C for 48 h.

### Sugar Analysis

Sugar content was determined by gas chromatography coupled to mass spectrometry (GC-MS). Sugars were extracted as described (Gómez-González et al., 2010) using 100 mg of lyophilized plant tissue and analyzed by GC-MS as described by Cerdán-Calero et al. (2012). Calibration curves from standards D-(+)-glucose, D-(−) fructose and D-(+)-sucrose (5, 10, 20, 30, 40, and 50 μg) were prepared for identification and quantification. In every case, 10 μg of xylitol was added as reference to the samples. GC-MS was performed on an Agilent Technologies GC system 7890A coupled to a mass spectrometer 5975C fitted with a column HP-5MS (30 m × 0.25 mm, Agilent). The temperature program was isothermal at 150°C for 3 min, followed by a 5°C/min gradient up to 210°C, a 15°C/min gradient up to 310°C, and isothermal for 2 min. The ionization potential was 70 eV, and spectra were recorded in low-resolution mode.

### Phytohormone Analysis

The following phytohormones were analyzed by using liquid chromatography coupled to mass spectrometry (LC/MS): abscisic acid (ABA), gibberellins (GA_3_ and GA_4_), indole-3-acetic acid (IAA), brassinolide (CS: castasterone), cytokinins [CKs: dihydrozeatin riboside, (DHZR), dihydrozeatin (DHZ), isopentenyladenine (Ip), isopentenyladenine riboside (iPR), trans-zeatin (tZ), trans-zeatin riboside (tZR)]. Phytohormones were extracted from 120 mg of lyophilized tissue (20–50 individuals, depending on the sampling time, 0, 24, and 216 h post-imbibition) as described by Pan et al. (2008). Phytohormones were separated and quantified by ultra-high performance liquid chromatography (UHPLC) in a 6460 Triple Quad LC/MS (Agilent Technologies) using the protocol described by Novák et al. (2008).

### RNA Extraction and cDNA Synthesis Analysis

Total RNA was extracted from 20 mg of embryos and germinated seeds by using the InviTrap^®^ Spin Plant RNA Mini Kit (Invitek), following the manufacturer’s directions with minor modifications (Echevarría-Zomeño et al., 2012). RNA quantitation was performed with the Qubit^®^ RNA Assay Kit in a Qubit^®^ 2.0 Fluorometer (Invitrogen) and RNA quality was checked electrophoretically (Agilent 2100 Bioanalyzer). Only high-quality RNAs with RIN values >8 and A260:A280 ratios of approximately 2.0 were used for subsequent experiments (Fleige and Pfaffl, 2006). cDNAs were generated from 1 μg of total RNA using the iScript cDNA synthesis kit (Bio-Rad) according to the manufacturer’s instructions. Reverse transcriptions were set up from RNA samples of identical concentrations in order to add the same volume to the RT reaction (Taylor and Mrkusich, 2014). An RNA “calibrator” with a known number of transcripts of the A170 gene was introduced in each experiment to guarantee the quality of the reverse-transcription, and to set the threshold in the different qRT-PCR plates (inter-plate calibrator).

### Primer Design and Sequencing

Sequences from *Q. ilex Dhn3*, *Gapdh*, *Sod1* and *Rblc genes*, or corresponding to *Fdh, Gst*, *GolS*, *Nadh6, Ocp3, Sdir1* and *Skp1* orthologs from different phylogenetically related species (preferentially *Quercus* > *Fagaceas* > *Fagales* > other plants) were obtained from the GenBank database^1^ (Supplementary Table S1). Alignments were performed by using the ClustalW software (MegAlign, DNASTAR Lasergene, v.6). Primer pairs were designed over conserved sequences and obtained using the Primer-BLAST tool of NCBI^2^. The proposed primer pairs were analyzed with the OLIGO Primer Analysis Software v 7.58 (Molecular Biology Insights, Inc.) and one pair for each gene, with high Tm and free from hairpin and duplex structures, was chosen. These primers were used to amplify synthesized cDNA from pooled *Q. ilex* RNAs and to obtain sequences from the species. PCR amplification was achieved by mixing 50 ng of *Q. ilex* cDNA with 0.75U of iTaq^TM^ DNA Polymerase (Bio-Rad) and following the manufacturer’s recommendations. Amplifications were carried out using an iCycler iQ Real-Time PCR System (Bio-Rad). PCR products were electrophoretically separated and visualized on agarose gels (2%) containing GelRed^TM^ (Biotium). The DNA bands of the predicted size were excised from the gels, purified (Wizard^®^ SV Gel and PCR Clean-Up System kit, Promega) and sequenced on an ABI PRISM^TM^ 3130 XL sequencer (Applied Biosystems). The identity of the trimmed sequences was confirmed using tBLASTx algorithm on the BLAST server at the NCBI databank, and the *Q. ilex* sequences were deposited in the GenBank Database (accession numbers in Supplementary Table S2). These sequences were used to design primers that exactly complemented the *Q. ilex* genes for the absolute quantification of transcript levels by real-time RT-PCR (qRT-PCR). To obtain a high specificity and a better performance, primers, free from hairpin and duplex structures, were required to have high *T*_m_ (≥70°C), and an optimal 3′-ΔG (≤−3 kcal/mol) value to be used in two-step PCR reactions. All primer pairs produced amplicons of the predicted size (Supplementary Table S2). All PCR products were further verified by nucleotide sequencing.

### qRT-PCR

Real-time PCR reactions were performed in quadruplicate with 50 ng cDNA per reaction, using an iCycler iQ thermocycler (Bio-Rad) and the iQ SYBR Green SuperMix (Bio-Rad), following the manufacturer’s directions. The amplification program consisted of one cycle at 95°C for 3 min, and 40 two-step amplification cycles at 95°C for 15 s and 68°C for 30 s, respectively. After 1 min at 95°C, a melting curve was obtained by following the fluorescence intensity during gradual cooling from 95 to 65°C.

To carry out absolute qRT-PCR, a calibration curve was constructed with an *in vitro* synthesized RNA, as previously detailed by Prieto-Alamo et al. (2003).

### Protein Immunoblot Analysis

Two hundred milligrams of plant material (corresponding to 20–50 embryos or seedlings) was employed for western blot analysis. Tissue was trichloroacetic–acetone–phenol extracted as previously described (Wang et al., 2006). The final pellet was suspended in 9 M urea containing 4% CHAPS, 0.5% Triton X-100 and 100 mM DTT, and insoluble material was eliminated by centrifugation. Protein concentrations were determined by the Bradford method (Bradford, 1976), using bovine albumin as standard. Proteins (25 μg) were resolved by SDS-PAGE, using 12% polyacrylamide gels (Mini-PROTEAN^®^ TGX Stain-Free^TM^ Precast Gels, Bio-Rad) with a Mini Protean Tetra-Cell (Bio-Rad). After electrophoresis, the gel image was captured and analyzed using a ChemiDoc^TM^ MP Imaging System (Bio-Rad). Proteins were transferred onto polyvinylidene difluoride (PVDF) membranes by using a Trans-Blot^®^ Turbo^TM^ Transfer System (Bio-Rad). After transfer, both gels and PVDF membranes were briefly rinsed with distilled water and the captured images were analyzed (ChemiDoc^TM^ MP Imaging System, Bio-Rad) to assess the quality of the transfers. Images were used to normalize band intensity after incubating membranes with the corresponding primary and secondary antibodies (Lee et al., 2005). PVDF membranes were blocked with 2% non-fat milk powder. Later, the membranes were incubated with rabbit antibodies raised against GAPDH (Sigma-Aldrich; dilution: 1:500), Dehydrin (Agrisera; dilution: 1:100) or RBCL (Agrisera; dilution: 1:100), for 1–3 h, washed and incubated with the secondary goat anti-rabbit IgG antibody (Sigma: A-3687, 1:2000), and conjugated to alkaline phosphatase for an additional 1 h. Image acquisitions and densitometric analyses were performed with the ChemiDoc MP Imaging system and ImageLab 4.1 software (Bio-Rad), respectively.

### Superoxide Dismutase Assays

Protein extracts for superoxide dismutase (SOD) assays were obtained from cryo homogenized tissue, by mixing 500 mg of each sample with 1 mL of 10 mM Tris–HCl buffer (pH 7.4), 5 mM DTT, 2 mM EDTA; 0.5% (v/v) Triton X-100, 5 mM ascorbic acid, 100 mM PMSF and 10% (w/v) PVPP. The mixtures were vortexed and sonicated four times (10 s at 6 W). Supernatants were collected by centrifugation (10000*g*, 10 min at 4°C) and used for enzyme assays. Protein concentrations were quantified by the Bradford method (Bradford, 1976), using bovine albumin as the standard. To preserve the enzymatic activity, the extracts were supplemented with 30% (w/v) sucrose and stored at −80°C until used, for no more than 1 week. SOD isoforms were separated on 10% non-denaturing polyacrylamide gels at 4°C, by using a mini protean electrophoresis unit (Bio-Rad, United States) and loading 20 μg of protein per well. After electrophoresis, the gels were stained for SOD activity as described by Weydert and Cullen (2010). The SOD isoforms were differentiated as described in Rowe et al. (1997).

### Statistical Analysis

In all the experiments, data from moisture, sugar, phytohormone, qRT-PCR, protein immunoblot, and SOD analyses were subjected to a univariate analysis of variance (ANOVA) and mean values were compared by Tukey’s test for *P* ≤ 0.05 with the software InStat v2.05/00 (GraphPad).

## Results and Discussion

### Germination Stages and Relative Water Content

**FIGURE 1:**
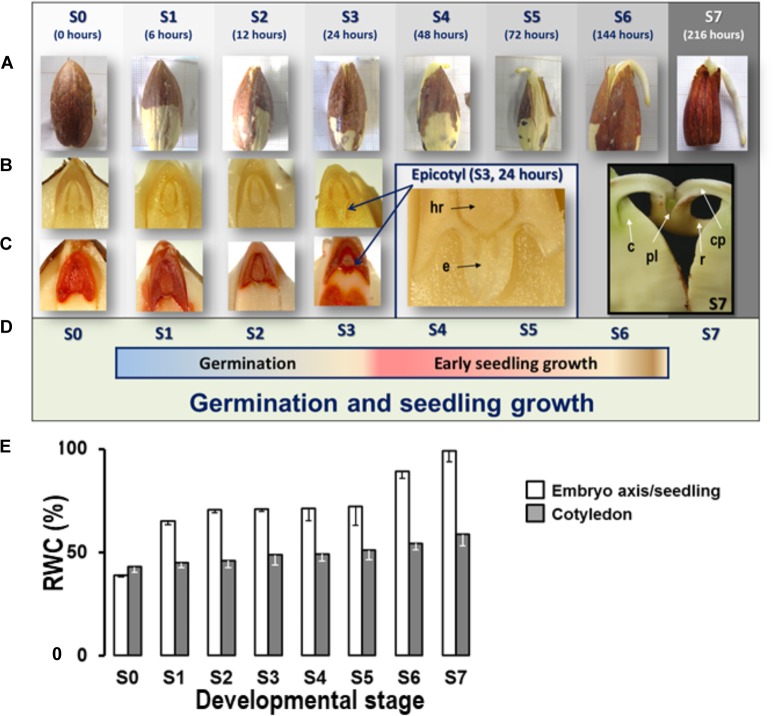
Morphology and physiology of *Quercus ilex* seeds germinating at different developmental stages. **(A)** Morphology of germinating acorns and young seedlings at each developmental stage; the relative time taken for low hydrated seeds to reach each stage is also shown; germinating dehulled seeds shows important morphological changes after imbibition at S2: testa rupture at 12 h of germination. S3: radicle emergence at 24 h; S5: epicotyl emergence from the embryonic axis; and S6: plumule emergence from differentiated cotyledonary petioles. **(B)** Seed sections showing the embryonic axes and the progressively differentiated parts of the embryo: hr, hypocotyle radicle; e, epicotyl; pl, plumule; c, cotyledon; cp, cotyledonary petiole; r, radicle; **(C)** TZ staining was used to facilitate the visualization of the morphological changes produced in the embryonic axes during germination. **(D)** Correspondence among the eight developmental stages and the phases of *Q. ilex* seed germination and seedling growth: germination (S0–S3) and early seedling growth (S4–S7). **(E)** Percentage of relative water content (RWC %) of complete germinating *Q. ilex* seeds or their cotyledons at the different developmental stages. The fresh (FW) and dried (DW) weights (70°C for 48 h) of 10–15 germinating acorns or seedlings were plotted per stage, with vertical bars representing ± SE of the mean. All mass measurements were made using an analytical scale, with precision of 0.0001 g. The relative water content (RWC) in germinating seeds was expressed as percentage of lost weight [(FW–DW) × 100] relative to fresh weight (FW). All values were rounded to the nearest milligram.

Seed germination is a complex process, comprising events from seed imbibition to radicle emergence. Morphologically, initiation of growth corresponds to radicle emergence; and subsequent development is generally defined as seedling growth (Bewley, 1997). In all the replicates, synchronized germination of 100% of the seeds was reached if they were previously dehulled. Eight developmental stages referred to as S0 to S7, were differentiated (Figure 1). Changes in morphology were observed during germination; at stage S2, the rupture of the testa was appreciable, with radicle emergence starting to be visible at stage S3, and plumule emergence from cotyledonary petioles at 216 h (S7) (Figures 1A–C). These changes indicate that the *Q. ilex* seed germination covers the first 24 h after imbibition, under our experimental conditions; followed by early seedling growth (from S4 to S7) (Figure 1D).

Relative water content was determined at the different developmental stages (Figure 1E), values for mature *Q. ilex* seeds were of 38%, which is in accordance with the data previously reported for *Quercus* spp. (i.e., Echevarría-Zomeño et al., 2009; Tilki, 2010). Then, the typical triphasic water uptake curve reported in germinating seeds (Finch-Savage and Leubner-Metzger, 2006; Rajjou et al., 2012; Bewley et al., 2013), was observed, including rapid initial water uptake (phase I, i.e., imbibition; S0–S2), followed by a plateau phase (phase II, S2–S5), and a further increase (phase III S5 and forward) (Figure 1E).

### Sucrose, Glucose, and Fructose Content

**FIGURE 2:**
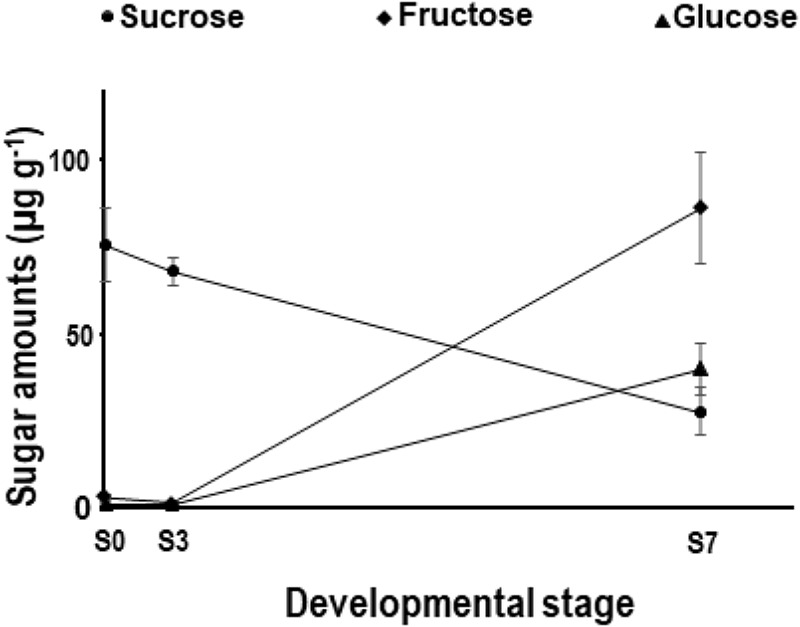
Sugar content in germinating *Q. ilex* seeds. Sucrose, glucose, and fructose content in the embryo axis at the S0, S3, and S7 stages were determined by GC-MS. Data are means ± SE of measurements made on 20–50 seeds per sampling time, grouped in three pools. Changes between the S3 and S7 stages were statistically significantly different at the *P* < 0.001 value (Student’s *t*-test).

Soluble carbohydrates have been proposed to play an important role in desiccation tolerance (Finch-Savage et al., 1996) and are associated with metabolic activity during germination and early seedling growth (Białecka and Kȩpczyński, 2007). Sucrose, fructose, and glucose were analyzed in embryos or seedlings at three stages, S0, S3, and S7, by means of GC-MS/MS. Soluble carbohydrate analysis in *Q. ilex* and other recalcitrant seeds during germination is scant in the literature. The level of sucrose (83 ± 0.4 μg g^−1^, <1% of DW; Figure 2) accumulated in the embryonic axis of *Q. ilex* seeds (S0 stage) was in the lowest range, even compared with other recalcitrant seeds (Steadman et al., 1996). Data reported for sucrose in orthodox seeds range from 33% in African oil bean (*Pentaclethra macrophylla*) to 0.3–3% in the seeds of some crops such as chick pea (*Cicer arietinum*) or black gram (*Vigna mungo*) (Black and Bewley, 2000), respectively. The amount of glucose or fructose in *Q. ilex* seed (3.0 ± 0.7 and 6.7 ± 0.7 μg g^−1^ DW, respectively) at S0 stage is very small, similar to that described for orthodox seeds by Black and Bewley (2000). Several factor are involved in the recalcitrant character of seeds (Pammenter and Berjak, 2014). In *Q. ilex* seeds the low content of these three sugars might contribute to this trait.

The levels of both monosaccharides increased dramatically (10–20-folds) during the late S7 stage and were accompanied by a statistically significant decrease in the amount of sucrose, from 83 ± 0.4 to 28 ± 6.6 μg g^−1^ DW. Similar changes have been reported in germinating *Glycine max* (Kuo et al., 1990) and *Moringa oleifera* (Tesfay et al., 2016) seeds. The germination and early seedling growth processes are characterized by a high metabolic activity in order to satisfy the energy required by them for development and growth (Nkang, 2002; Rajjou et al., 2012). In support of this hypothesis, a decrease in sucrose and an increase in monosaccharides was observed at stages S3 to S7.

### Phytohormone Profiling

The ABA content in mature *Q. ilex* embryo axes (6.4 ± 1.0 ng g^−1^ DW) reported here was similar to that described for the embryo axis of *Q. robur* (Prewein et al., 2006) and other recalcitrant seeds (Pieruzzi et al., 2011). However, this value is considerably lower than those reported for orthodox seeds of *Arabidopsis thaliana* (140 ng g^−1^ DW) (Liu et al., 2009) or *Solanum lycopersicum* (50 ng g^−1^ DW) (Yang et al., 2014). The desiccation sensitivity and absence of dormancy in *Q. ilex* seeds are probably related to their low ABA levels, emphasizing the role of this phytohormone in preventing premature germination and desiccation tolerance acquisition (Ali-Rachedi et al., 2004). In fact, in orthodox species *A. thaliana*, *aba* mutants unable to synthesize ABA and ABA signaling mutants are less desiccation tolerant and less dormant (Parcy et al., 1994; Lefebvre et al., 2006; Nakashima et al., 2009).

**FIGURE 3:**
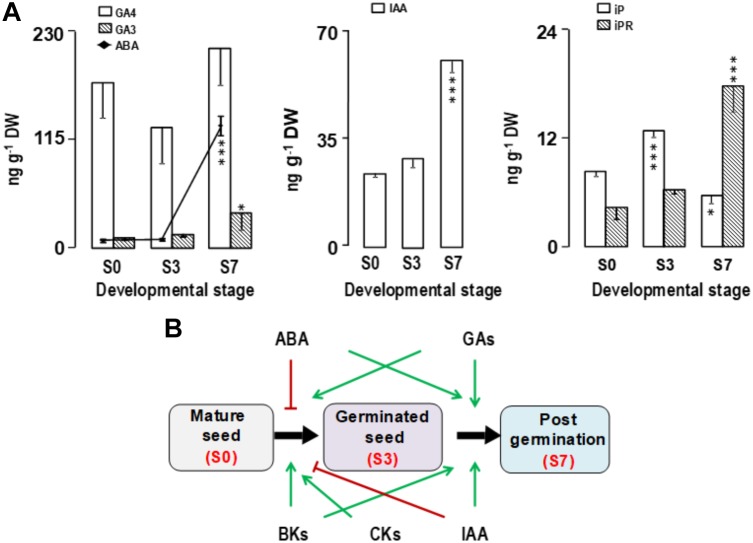
**(A)** Changes in the endogenous concentrations of phytohormones in *Q. ilex* embryo axis and seedling during germination and post-germination stages. The levels of abscisic acid (ABA), gibberellins (GA3, GA4), auxin (IAA), and cytokinins (iP, iPR,) were determined by using GC-MS/MS. Data are means ± SE of measurements made on 20–50 seeds per sampling time, grouped in three pools. Statistical significance was determined using the one-way ANOVA Tukey’s test. The *P*-values are given as ^∗^*P* < 0.05 level and ^∗∗∗^*P* < 0.001 level. **(B)** Phytohormone interactions during germination and seedling development.

The amount of ABA was constant during the germination process (S0 to S3), although the ABA amount increased considerably (>20-fold) during early seedling growth (from S3 to S7; Figure 3A). This fact has also been described for the recalcitrant species *Araucaria angustifolia* (Pieruzzi et al., 2011). The high levels of ABA at the S7 stage might be related to the development of *Q. ilex* seedlings. Recent studies indicated a positive role for ABA in promoting root meristem maintenance and root growth in *Arabidopsis*, *Zea mays*, *Vicia faba* and *Helianthus annuus*, but the underlying molecular mechanisms remain unknown (Finkelstein et al., 2002; Zhang et al., 2010; McAdam et al., 2016).

The level of GAs is usually undetectable in orthodox non-germinating seeds but its accumulation is a prerequisite for dormancy breaking (Toh et al., 2008; Chen et al., 2010; Weitbrecht et al., 2011), with GA_4_ being the major endogenous active GA in germinating seeds and shoots (Ogawa and Hanada, 2003). Related to the absence of dormancy, *Q. ilex* seeds showed high levels of gibberellins throughout all the developmental stages surveyed, from mature seeds (S0) to early growth seedlings (S7) (Figure 3A). GA_4_ was the major gibberellin in *Q. ilex* seeds (174.8 ± 37.1 ng/g DW), with much higher values than those found in germinated orthodox seeds (Ogawa and Hanada, 2003; Chen et al., 2010), and remained unchanged throughout the course of the experiment. A 15 times lower content was found in mature seeds for GA_3_ (10.6 ± 2.9 ng/g DW), that increased up to 3-times (37.0 ± 18.1 ng/g DW) in S7, early growth seedlings. An increase in GA_3_ has also been reported in orthodox seeds of *Phellodendron amurense* (Chen et al., 2010) and recalcitrant *Avicennia marina* (Farrant et al., 1993), although different amounts of the hormone at the mature seed stage were provided. The significant increase of GA_3_ in early seedling growth of *Q. ilex* could be related to growth promotion, a biological function described for active GAs (Zhang, 2007; Vishal and Kumar, 2018).

Small amounts of ABA and large ones of active GAs are required for seed germination (Shu et al., 2016). GA does not appear to control dormancy *per se* but acts by stimulating radicle emergence (Bewley, 1997). Our findings in the embryonic axes of mature *Q. ilex* seeds showed this pattern, therefore indicating that *Q. ilex* seeds have the hormone balance necessary to proceed to germination at the time of shedding.

This ABA and GA pattern in mature seeds are one explanation of the non-dormant character of *Q. ilex* seeds. During seedling growth (from S3 to S7; Figure 3A), increases in ABA and GAs were observed; the high amount of ABA in seedling (S7) of *Q. ilex* could be necessary for the root development. This result is in accordance with that reported by McAdam et al. (2016), where ABA-biosynthetic mutants of *Pisum sativum* and *S. lycopersicum* showed reduced root biomass highlighting the importance of ABA in root development.

Indole-3-acetic acid is involved in seed developmental processes, interacting with other hormones and controlling dormancy and germination (Figure 3B; Finkelstein et al., 2008; Miransari and Smith, 2014; Shu et al., 2016). We found an increase in the amount of IAA during the experiment (Figure 3A), which was expected, since IAA has often been implicated in plant growth (Santner et al., 2009) and root development (Tromas and Perrot-Rechenmann, 2010). The IAA levels in *Q. ilex* (23.9 ± 1.4 ng g^−1^ DW) mature seeds were lower than those reported in *A. marina* (500 ng g^−1^ DW) (Farrant et al., 1993). Similar IAA increases during germination and early seedling development to those reported here have been described for several other recalcitrant seeds (Farrant et al., 1993; Pieruzzi et al., 2011). The increase in IAA and ABA content during *Q. ilex* seedling growth supports the idea that there is crosstalk between IAA and ABA (Liu et al., 2007; Nguyen et al., 2016), and that both hormones are involved in the processes occurring during *Q. ilex* seed germination and seedling growth (Figure 3B).

We also analyzed the levels of some cytokinins and the brassinolide castasterone (CS), in *Q. ilex* embryo axes and seedlings during germination and early seedling growth. Cytokinins play an important role in promoting cell division and elongation in the embryo and providing positional information for the developing embryo (Leubner-Metzger, 2006). It is also known that cytokinins antagonize ABA during seed germination (Wang et al., 2011). Among the CKs and brassinolide measured here (tZ, iP, DHZ, iPR, tZR and DHZR, and CS), only iP and iPR showed significant differences during the stages studied (*P* < 0.001; Figure 3A). The tZ, tZR, DZ and DZR levels (data not shown) do not shown significant variation during germination as observed in recalcitrant seeds of *A. marina* (Farrant et al., 1993), and the most abundant cytokinin was tZR. In orthodox seeds the predominant free cytokinin was DHZ-in *Medicago sativa* L. while cZ was predominant in *Z. mays* and *Avena sativa.* Their levels shown some variation during germination (Stirk et al., 2012).

The iP content in *Q. ilex* seeds/seedlings increased from 8.5 ± 0.5 to 12.6 ± 0.6 ng g^−1^ DW during germination (S0–S3), and then decreased at S7 stage (Figure 3A), reaching lower levels than in the S0 stage. The *Q. ilex* seed content of iP was lower than that described in other recalcitrant species, but showed a similar time-course profile (Farrant et al., 1993; Farrant and Moore, 2011). In contrast to iP, iPR exhibited a continuous increase during germination and post-germination stages (from 4.2 ± 1.3 ng g^−1^ DW in S0 to 17.7 ± 2.9 ng g^−1^ DW in S7). To our knowledge, this is the first study reporting the time-course increase in iPR in this type of seed and demonstrating that, in recalcitrant *Q. ilex* seeds, the increase in-dormancy-releasing hormones accompanies germination, and that the mature *Q. ilex* seed has the phytohormone balance necessary for germination.

Our data support a model for non-dormant recalcitrant seeds of *Q. ilex*, low ABA content is insufficient to prevent premature germination, while high levels of GAs, auxin, and CKs promote germination.

### Targeted Transcriptional Profiling

A targeted transcriptional analysis was performed on 11 genes of relevance in seed viability and germination, whose expression is expected to be different between orthodox and recalcitrant species (Supplementary Table S2). The list included genes related to desiccation tolerance (Nishizawa et al., 2008; Hanin et al., 2011), ABA-signaling regulation (Cutler et al., 2010), metabolism (Muñoz-Bertomeu et al., 2009; David et al., 2010), and oxidative stress (Giannopolitis and Ries, 1977; Roxas et al., 1997).

**FIGURE 4:**
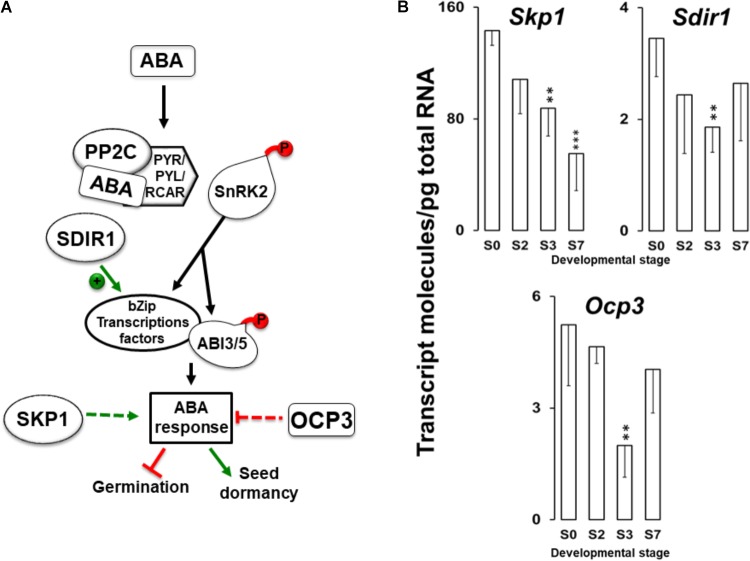
**(A)** A simplified working model of the ABA signaling pathway controlling the germination process indicating the participation of the *Skp1*, *Sdir1*, and *Ocp3* gene products. **(B)** Absolute quantitation of *Skp1*, *Sdir1*, and *Ocp3* transcript molecules in embryo axis tissue isolated from non-imbibed seed (S0) or after 10 h (S2), 24 h (S3), and 216 h (S7) of germination. Transcript data are the means ± SD of transcript molecules/pg of total RNA from three biological replicates made in quadruplicate. Each biological replicate was a pool generated by mixing equal amounts of homogenized tissue from 10 to 30 embryo axis of the same sampling time. Statistical significances were determined by a one-way ANOVA. Differences between S0 samples and between each other were statistically significant at the level ^∗∗^*P* < 0.01 and ^∗∗∗^*P* < 0.001. Dashed lines indicate that multiple stages are included.

We analyzed *Skp1*, *Sdir1* and *Ocp3* genes coding for regulatory elements involved in the ABA signaling pathways, among which *Ocp3* is a negative regulator (Ramírez et al., 2009) while, *Skp1* and *Sdir* promote ABA effects (Zhang et al., 2007; Hu et al., 2013; Figure 4A). It is well established that ABA-dependent signaling is a main response to drought, salt stresses, and the maintenance of seed dormancy (Cutler et al., 2010 and ref. therein).

In a previous study, overexpression of *Skp1* led to a delay in germination, improved drought tolerance, and root growth inhibition in the presence of ABA (Li et al., 2012). Values for the *Skp1* transcript decreased as the germination and seedling growth progressed (from S0 to S7 stages) (Figure 4B), in parallel with the rise in ABA levels, (Figure 3A). Therefore, the decrease in *Skp1* expression from S0 to S3 could facilitate germination, and the continued decrease in *Skp1* expression continues to S7 stage might promote root growth.

In addition to SKP1, many other E3 ligases have been found to be involved in ABA responses. The RING type E3 Salt and Drought Induced RING Finger 1 (SDIR1) acts as an active RING-type E3 ubiquitin ligase, upstream of ABA-responsive transcription factors, in a feedback mechanism that enhances the ABA-signal (Lyzenga and Stone, 2012). *Sdir1* expression has been reported to be upregulated by drought and salt stress, but not by ABA. Overexpression of *Sdir1* leads to ABA hypersensitivity and drought tolerance (Zhang et al., 2007). In *Q. ilex* samples, *Sdir1* transcript values decreased ∼2-fold from S0 (3.5 molecules/pg total RNA) to S3 (1.8 molecules/pg total RNA), resulting in a promotion of germination. A slight increase in *Sdir1* expression was observed at the S7 stage (Figure 4B), but this expression level was slightly lower than the S0 stage. This result suggests that *Sdir1* could be involved in some extent in seedling development.

The *Ocp3* mRNAs diminished twofold during the germination (S0 to S3, from 5.2 to 2.3 molecules/pg total RNA) and increased thereafter during the post-germination phase by up to 3 molecules/pg total RNA at S7 stage. OCP3 is a member of the homeobox transcription factor family, and is considered as a negative regulator of the early response of the plant to drought stress since the *Ocp3* loss of function yields a hyper-susceptibility to the ABA hormone in *Arabidopsis*, but does not affect germination rate (Ramírez et al., 2009). Considering that *Ocp3* is a negative regulator of ABA signaling, its expression was unexpectedly decreased at S3 stage where ABA response should be lower to promote germination and there is no dehydration stress due to the imbibition of seeds. This result suggests that *Ocp3* has little or no effect on modulation of ABA response during the germination and rehydration process.

**FIGURE 5:**
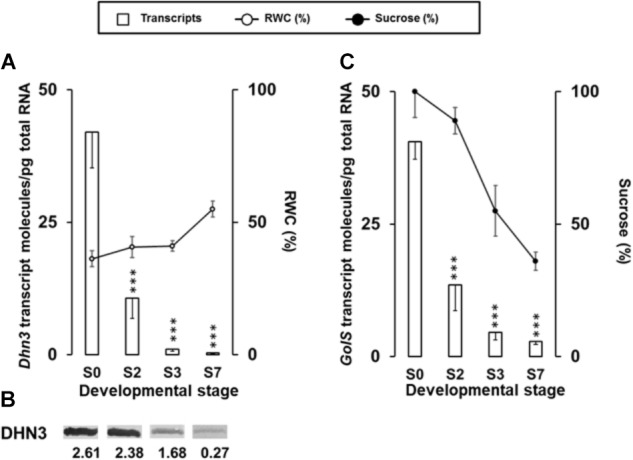
**(A)** Absolute quantitation of *Dhn3* transcript molecules in embryo axis tissue isolated from non-imbibed seed (S0) or after 10 h (S2), 24 h (S3), and 216 h (S7) of germination, compared with the relative water content (RWC) in germinating seeds. Transcript data are the means ± SD of transcript molecules/pg of total RNA from three biological replicates made in quadruplicate. Each biological replicate was a pool generated by mixing equal amounts of homogenized tissue from 10 to 30 embryo axis of the same sampling time. Statistical significances were determined by a one-way ANOVA. Differences between S0 samples and each other were all statistically significant (^∗∗∗^*P* < 0.001). RWC data, expressed as percentage of lost weight relative to fresh weight, correspond to those in Figure 1E and are included for correlation with *Dhn3* transcript amount. **(B)** Western blotting of DHN3 protein in *Q. ilex* seeds samples. Proteins were extracted from the same pools used in the transcriptional analysis. Numbers indicate the arbitrary Western blotting signal intensities normalized to the total protein contents, using Stain-Free Technology for total protein quantification. **(C)** Absolute quantitation of *GolS* transcript amounts in the same samples S0, S2, S3, and S7 and conditions described in panel **(A)**, compared with the sucrose content in germinating seeds. Sucrose data correspond to those in Figure 2 and included for correlation with *GolS* transcript levels.

Partial desiccation of up to 38% RWC takes place during maturation of *Q. ilex* seeds so some dehydration stress is expected. This can be confirmed by the presence of dehydration-responsive genes such as *Dhn3* and *GolS*. Figure 5 shows changes in *Dhn3* and *GolS* transcript abundance throughout the different developmental stages. Both are ABA-dependent dehydration-responsive genes, whose protein products protect plant proteins and membranes from water loss and help to maintain cell integrity during seed desiccation. Dehydrin DHN3 is a Group II Late Embryogenesis Abundant (LEA) family member, acting as a potent chaperone under dehydration stress conditions (Eriksson and Harryson, 2011). The accumulation of dehydrins was also reported previously in the recalcitrant *Q. robur* seeds (Šunderlíková et al., 2009), so their presence, at least at certain levels, cannot be correlated with desiccation tolerance and the orthodox character. *Dhn3* transcript abundance dropped dramatically and constantly (>120-fold) from the S0 stage up to the end of the experiment (S7), and showed an inverse pattern with the RWC (Figure 5A). That decrease was also observed for the DHN3 protein, as was revealed by western blot assay (Figure 5B).

*Gols* is an enzyme involved in the synthesis of the raffinose series oligosaccharides, which are osmoregulators associated with seed desiccation tolerance (Li et al., 2011). We analyzed the *GolS* mRNA pattern during germination and seedling growth (Figure 5C). Transcripts accumulated at high levels (>40 transcript/pg total RNA) in mature *Q. ilex* embryo axes (S0), dramatically dropped (>14-fold decrease) after imbibition (S2–S7), changing in parallel with the relative sucrose content in germinating seeds (Figure 5C). A decrease in the expression of *GolS* mRNA during germination process has been reported in chickpea (*C. arietinum*). The raffinose series oligosaccharides have also been reported as ROS scavengers protecting cellular components from oxidative stress (Nishizawa et al., 2008). Therefore, they might improve seed vigor and longevity through limiting the age-induced excess ROS and subsequent lipid peroxidation (Salvi et al., 2016).

During seed maturation in many species (mostly orthodox seeds), there are two peaks of ABA accumulation: the first is maternally derived and promotes reserve accumulation and inhibits vivipary, and the second has embryonic origin and is important for LEA synthesis, desiccation tolerance, and dormancy (Finkelstein et al., 2002). In the recalcitrant *Q. robur* seeds, a peak of ABA accumulation was observed at the end of the maturation process (Prewein et al., 2006). This accumulation of ABA could occur during *Q. ilex* seed maturation, leading to the expression of *Sdir1*, *Skp1*, *Dhn3*, and *GolS* which are present in the mature seeds (S0). Although these genes can contribute to the tolerance of partial desiccation of *Q. ilex* seed, their accumulation levels together with the low ABA, sucrose, glucose, and fructose content are not sufficient to confer orthodox seed character. Because the ABA signaling pathway is involved in acquisition of dormancy and desiccation tolerance and both occur during seed development, it is difficult to evaluate the contribution of *Sdir1*, *Skp1*, and *Ocp3* in dormancy on the one hand and in desiccation tolerance for the other hand, at least in our experimental conditions.

**FIGURE 6:**
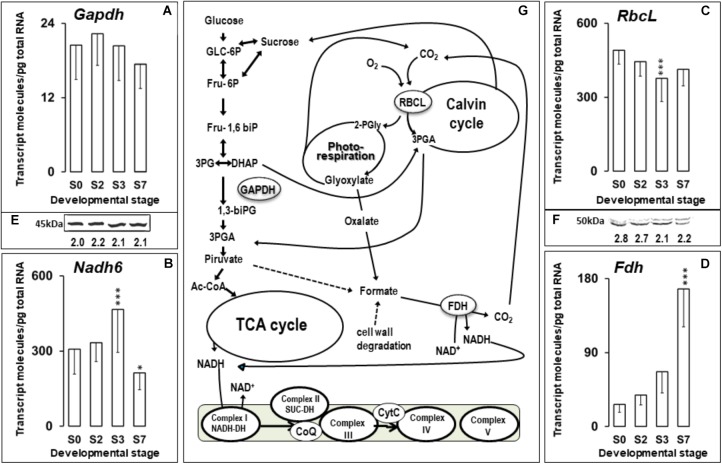
**(A–D)** Absolute quantitation of *Gapdh*, *Nadh6*, *RbcL*, and *Fdh* transcript molecules in embryo axis tissue isolated from non-imbibed seed (S0) or after 10 h (S2), 24 h (S3), and 216 h (S7) of germination. Transcript data are the means ± SD of transcript molecules/pg of total RNA from three biological replicates made in quadruplicate. Each biological replicate was a pool generated by mixing equal amounts of homogenized tissue from 10 to 30 embryo axis of the same sampling time. Statistical significance was determined by a one-way ANOVA. Differences between S0 samples and between each other were statistically significant at the level ^∗^*P* < 0.05; ^∗∗∗^*P* < 0.001. **(E,F)** Western blotting of GAPDH and RBCL proteins in *Q. ilex* seed samples. Proteins were extracted from the same pools used in the transcriptional analysis. Numbers indicate the arbitrary Western blotting signal intensities normalized to the total protein contents, using Stain-Free Technology for total protein quantification. **(G)** A simplified working model of some metabolic pathways indicating the participation of the *Gapdh*, *Nadh6*, *RbcL* and *Fdh* gene products.

A very active metabolism is a characteristic of germination and growth processes in order to satisfy the high energy and biomolecule demand of the growing tissues. In orthodox seeds, germination represents a switch from quiescence in mature seeds to a metabolism reactivation under a low oxygen concentration in germinating ones (Logan et al., 2001). In contrast, in recalcitrant seeds, maturation and germination is continuous, with an active metabolism also after shedding (Dias et al., 2010; Sghaier-Hammami et al., 2016). Figure 6 shows the variation in the transcript abundance of four genes representing metabolic activity, *Gapdh*, *Nadh6*, *Rbcl*, and *Fdh*, throughout the germination and early post-germination stages. *Gapdh* transcripts and proteins remained almost constant during germination and early post-germination stages of *Q. ilex* seeds (Figures 6A,E). Recent studies have shown that GAPDH has multiple functions independently of its role in energy metabolism. An increased GAPDH gene expression and enzymatic function is associated with cell proliferation, transcriptional, and posttranscriptional gene regulation, vesicular transport, receptor-mediated cell signaling, chromatin structure and the maintenance of DNA integrity (O’Halloran and Culotta, 2000; Aoki et al., 2006). *Nadh6* transcript numbers peaked early during acorn germination (Figure 6B), coinciding with radicle emergence (S3), and dropped later to the levels found in mature seeds. A similar increase in the abundance of *Nadh6* and other transcripts encoding mitochondrial proteins has been reported for *Arabidopsis* during the maturation of the mitochondria that occurs after imbibition (Howell et al., 2009). These changes were correlated with the decrease in sucrose (Figure 2) at germination (S0 to S3 steps) and the increases in glucose and fructose levels at post-germination (S3 to S7) stages. This indicated a modulation of the sugar catabolism and the adaptation to the energy and carbon demands by the developing tissues, similarly to those described for orthodox seeds (Downie and Bewley, 2000; Nkang, 2002).

The controversial presence of ribulose-1,5-bisphosphate carboxylase/oxygenase (RuBisCO) in non-photosynthetic tissue, such as seeds (Sghaier-Hammami et al., 2016) and roots (Simova-Stoilova et al., 2015), has been proven by proteomics in *Q. ilex* seeds and confirmed by data presented here on transcript abundance of the *RbcL* gene that codes for the RuBisCO large subunit. Speculating on the physiological role of the RuBisCO enzyme, it can be proposed as being implicated in the re-assimilating CO_2_ from decarboxylation reactions. In *Q. ilex* seeds, *RbcL* transcript abundance provided one of the highest values for the genes analyzed (∼400 molecules/ng total RNA) (Figure 6C). Transcription of *RbcL* has been shown to be repressed by soluble sugars (Kersten et al., 2006), which might explain the decrease in the *RbcL* transcript and protein levels during *Q. ilex* germination, when the soluble sugars increase (Figures 2, 6C,F).

The *Fdh* mRNA levels (Figure 6D) showed a constant increase throughout the different developmental stages studied here, becoming an abundant transcript, with more than 150 molecules/pg total RNA, at the end of the experiment. FDH is one of the enzymes whose gene is dependent on ABA (Alekseeva et al., 2011). The increase in endogenous concentrations of this phytohormone in *Q. ilex* seedlings during post-germination stages (Figure 3A) might explain this dramatic increase in *Fdh* mRNA.

**FIGURE 7:**
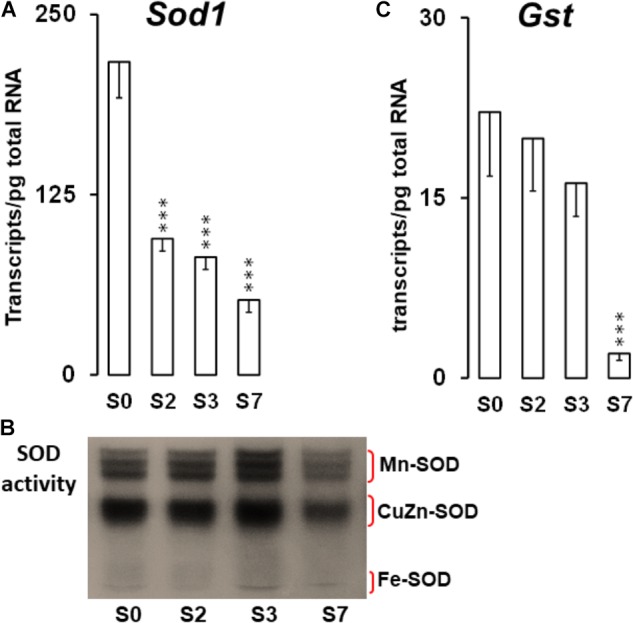
**(A,C)** Absolute quantitation of *Sod* and *Gst* transcript molecules in embryo axis tissue isolated from non-imbibed seed (S0) or after 10 h (S2), 24 h (S3), and 216 h (S7) of germination. Transcript data are the means ± SD of transcript molecules/pg of total RNA from three biological replicates made in quadruplicate. Each biological replicate was a pool generated by mixing equal amounts of homogenized tissue from 10 to 30 embryo axis of the same sampling time. Statistically significant differences between S0 samples were determined by a one-way ANOVA (^∗∗∗^*P* < 0.001). **(B)** SOD activity gel assay showing the SOD protein profiles on native PAGE of different *Q. ilex* seeds germination stages. Proteins were extracted from the same pools used in the transcript analysis. A 20 μg sample of total soluble proteins was loaded onto a 10% acrylamide gel. The image of the gel was inverted to obtain a better visualization of the bands.

Reactive oxygen species, exert a dual effect on germination, acting as messengers and causing oxidative damage to cell macromolecules and structures (Bailly et al., 2008). In orthodox seeds, elevated rates of ROS production upon seed imbibition has been suggested to be involved in cell wall loosening, and in defense of emerging seedlings against pathogens (Roach et al., 2010), and are necessary for proceeding to germination (Bailly et al., 2008). To keep ROS in balance, different tightly regulated mechanisms do exist, with redox enzymes acting as scavengers playing an important role. The transcript abundance of two of them, superoxide dismutase (SOD1) and glutathione-S-transferase (GST), was analyzed in the present work. *Sod1* transcriptional profile (Figure 7A) shows a significant decrease in the amount of *Sod1* mRNA molecules throughout the germination process. The decrease in the number of *Sod1* transcripts was initiated at S2 stage and might contribute to the drop in SOD1 activity detected later (S7 stage). These results suggest that ROS scavenging mechanisms have been activated during recalcitrant seed maturation, leading to the accumulation of SOD, one of the enzymes implicated in the balance of ROS, and allowing progression to germination (Barba-Espin et al., 2010). Three types of SODs have been classified on the basis of the metal present at the catalytic site: Cu/Zn-SOD (SOD1, located in the cytoplasm and chloroplasts), Mn-SOD (in the mitochondrial matrix and peroxisomes), and Fe-SOD (in chloroplasts and cytoplasm) (Redgwell and Fry, 1993; Myouga et al., 2008). By means of an *in gel* enzyme activity assay (Weydert and Cullen, 2010) and the SOD-inhibitors H_2_O_2_ and KCN, we identified three MnSOD isoforms (resistant to H_2_O_2_ and KCN), a single Cu/ZnSOD (sensitive to H_2_O_2_ and KCN) and a single FeSOD (resistant to KCN and sensitive to H_2_O_2_) in *Q. ilex* seeds (Figure 7B and Supplementary Figure S3). Bands of the five isoforms were present in all the stages studied.

Plant GSTs are a super-family of proteins of a divergent sequence but conserved structure, which catalyze the conjugation of electrophilic xenobiotic substrates with the tripeptide glutathione (GSH), and which are selectively stress-inducible. Some GSTs can also act as glutathione peroxidases, protecting cells from oxygen toxicity (Binder et al., 2004). Our results revealed that the abundance of *Gst* transcripts decreases during germination in *Q. ilex* acorns (Figure 7C). Our findings also suggest, that the accumulation of SOD and GST transcripts, implicated in ROS balance in mature seeds, could be necessary to protect the tissue from ROS damage with other antioxidant enzyme such as catalase, peroxidase, etc. Although these enzymes were not analyzed in this work, their activity was previously described during recalcitrant seed germination (Gumilevskaya and Azarkovich, 2007). ROS also act as messengers in signal transduction pathways during germination (Bailly et al., 2008; Wojtyla et al., 2016).

## Conclusion

The germination and early seedling growth process, and the non-orthodox, non-dormant character of *Q. ilex* seeds, have been partially characterized at the molecular level.

The main differences with orthodox seeds are already established in mature ones, and are manifested at the germination (S1–S3) stage. Once germinated, the patterns are quite similar between both types of seeds.

The hormonal balance (low ABA and high GA, IAA and cytokinin content) and the presence of enzyme machinery ensuring an active metabolism (i.e., GAPDH and NADH) seem to contribute to the non-dormant character.

Although desiccation tolerance genes were expressed, their accumulation level together with the low ABA, sucrose, glucose, and fructose content are not sufficient to confer orthodox seed character.

The high transcript levels of two ROS scavenging enzymes found in mature seeds, suggest that these enzymes are important in maintaining ROS balance in *Q. ilex* seeds.

## Author Contributions

JJ-N conceived the work, examined, and evaluated the data. MR-R and AA-Y carried out the gene expression and protein immunoblot assays, and measurements of enzyme activities. NA examined and evaluated the gene expression data. AG-S performed the sugar analysis. MM conducted the hormone analysis. The manuscript was written by JJ-N and MR-R, and finally approved by the rest of the authors.

## Conflict of Interest Statement

The authors declare that the research was conducted in the absence of any commercial or financial relationships that could be construed as a potential conflict of interest.

## Footnotes

1 http://www.ncbi.nlm.nih.gov

2 https://www.ncbi.nlm.nih.gov/tools/primer-blast/

## Supplementary Material

FIGURE S1 Process to acorn germination. **(A)** Acorn with pericarp, **(B)** acorn peeled, **(C)** acorn cut at the distal end and **(D)** boxes contain filter paper, perlite, and a germinated acorn.

FIGURE S2 Morphological aspects of acorns and seeds at different stages. In brackets the approximate time in hours post-imbibition to obtain the indicated stages.

FIGURE S3 SOD in-gel activity assay of embryo axis and shoot seedling of *Q. ilex*. **(A)** Inhibitor test for SOD isoforms was conducted by application of 2 mM KCN or 5 mM H_2_O_2_ prior to activity staining. **(B)** SOD isoforms are labeled S1–S5 in order of increasing mobility.

TABLE S1 Primers used in this work for the determination of *Q. ilex* sequences. Primers based on orthologous sequences were designed to obtain partial coding sequences of *Q. ilex* genes.

TABLE S2 Primers used for absolute quantification by real-time PCR of *Q. ilex* transcripts.
