# Supplementary figures and images for: Germination and Early Seedling Development in Quercus ilex Recalcitrant and Non-dormant Seeds: Targeted Transcriptional, Hormonal, and Sugar Analysis
Source: Front Plant Sci. 2018 Oct 22;9:1508. doi: 10.3389/fpls.2018.01508 (PMC6204751; doi:10.3389/fpls.2018.01508)

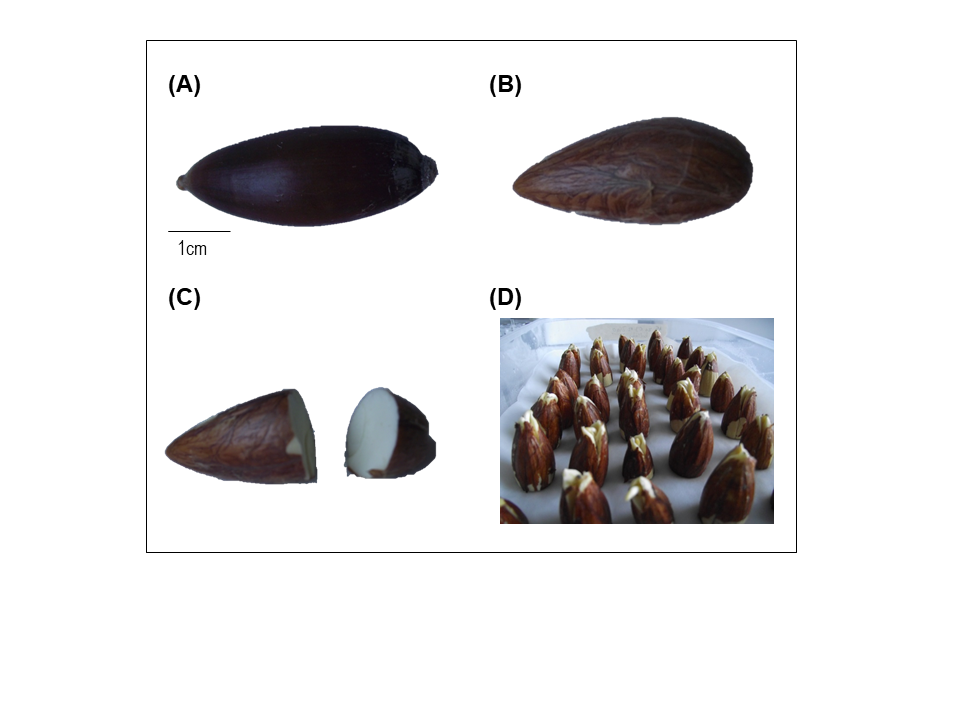

Supplement: FIGURE S1 — Process to acorn germination. (A) Acorn with pericarp, (B) acorn peeled, (C) acorn cut at the distal end and (D) boxes contain filter paper, perlite, and a germinated acorn. [file Image_1.TIF]

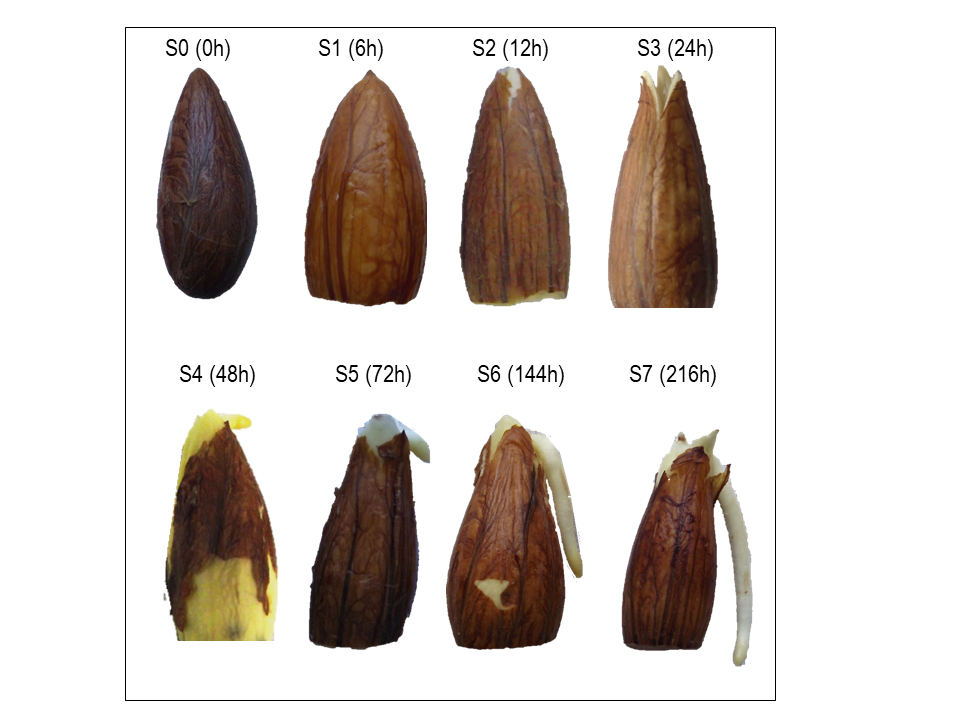

Supplement: FIGURE S2 — Morphological aspects of acorns and seeds at different stages. In brackets the approximate time in hours post-imbibition to obtain the indicated stages. [file Image_2.TIF]

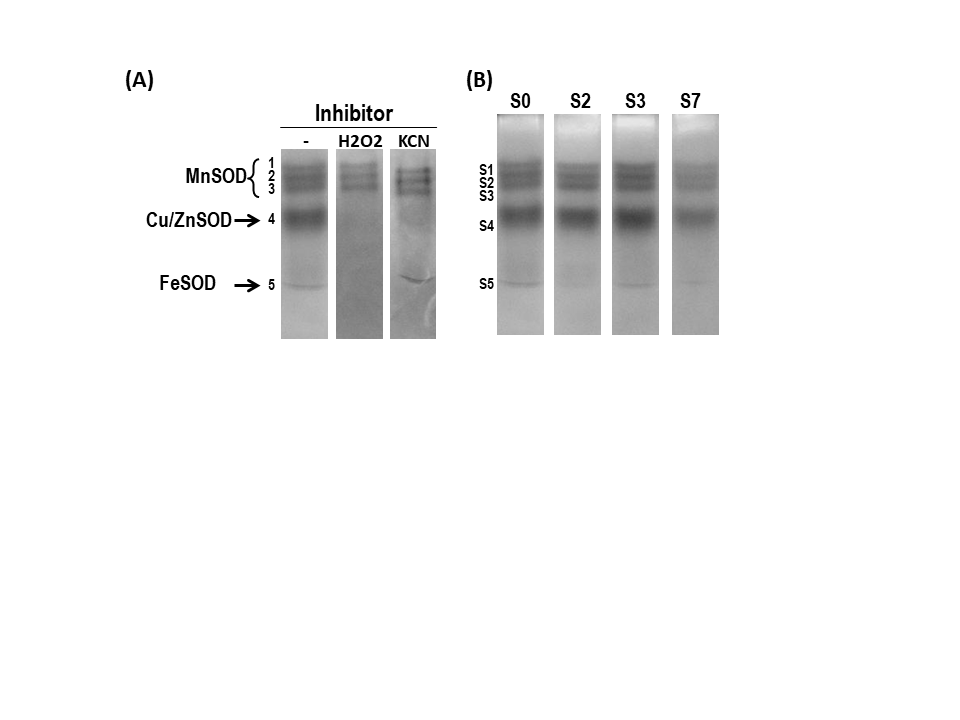

Supplement: FIGURE S3 — SOD in-gel activity assay of embryo axis and shoot seedling of Q. ilex. (A) Inhibitor test for SOD isoforms was conducted by application of 2 mM KCN or 5 mM H2O2 prior to activity staining. (B) SOD isoforms are labeled S1–S5 in order of increasing mobility. [file Image_3.TIF]
